# Treatment Effects of Genital Mycoplasma Species in High-Risk Pregnant Individuals: A Retrospective Cohort Study

**DOI:** 10.7759/cureus.98095

**Published:** 2025-11-29

**Authors:** Takashi Yoshimasu, Tasuku Inao, Tetsuya Kawakita

**Affiliations:** 1 Department of Biostatistics, Graduate School of Medicine, Hokkaido University, Sapporo, JPN; 2 Department of Obstetrics and Gynecology, Macon and Joan Brock Virginia Health Sciences at Old Dominion University (ODU), Norfolk, USA

**Keywords:** cohort study, genital mycoplasma, preterm birth, spontaneous abortion, vaginal infection

## Abstract

Objective

We aimed to estimate the causal effect of treatment for genital *Mycoplasma* species on preterm birth (PTB) and spontaneous abortion (SA). We also evaluated treatment effects separately for cases with co-infection of *Ureaplasma* and *Mycoplasma *species.

Methods

This was a retrospective observational cohort study at a single center. Singleton mothers with one or more risk factors for PTBs were screened and treated for genital *Mycoplasma* species. The treatment was repeated based on polymerase chain reaction (PCR) results after each course. We applied inverse probability treatment weighting, and risk differences (RDs) of the treatment effect on pregnancy outcomes were estimated with the generalized estimating equations. A combination of PTB and SA was used as the primary outcome. Secondary analysis was conducted, focusing on treatment for co-infection of both *Mycoplasma* and *Ureaplasma* species.

Results

From January 2014 to December 2020, 696 individuals tested positive for genital *Mycoplasma *species. Of the individuals, 566 were treated with azithromycin, whereas 62 were not. Among them, 269 received a second treatment, whereas 40 did not. The first treatment effect on PTB and SA was RD (95% CI) of 0.037 (-0.104, 0.178), and the second treatment effect was RD (95% CI) of -0.049 (-0.220, 0.123). When focusing on co-infection of *Ureaplasma* and *Mycoplasma*, the first effect was RD (95% CI) of -0.005 (-0.274, 0.264), and the second treatment effect was RD (95% CI) of -0.545 (-0.739, -0.350).

Conclusions

Routine treatment for genital *Mycoplasma* species* *is not recommended. However, the results suggest potential benefits in treating co-infections.

## Introduction

Preterm birth (PTB) accounts for 9.9% of all births globally and is one of the major risks for neonatal mortality under five years of age [1], and maternal infections are responsible for 25-40% of all PTBs [2]. Additionally, the risk of spontaneous abortion (SA) is 15.3% of all recognized pregnancies globally [3]. Maternal infections are associated with SA in 15% of cases during early gestational weeks (~12 weeks) and in 66% during late gestational weeks (12~24 weeks) [4].

Genital *Mycoplasma* species, including *Ureaplasma (U.) urealyticum, U. parvum, Mycoplasma (M.) hominis,* and *M. genitalium*, belong to the class Mollicutes and are considered part of normal genital flora, with a colonization rate of 40-80% [2,5,6]. They are associated with adverse pregnancy outcomes, including PTB, SA, chorioamnionitis, preterm premature rupture of membranes (PPROM) [7-10], neonatal pneumonia, and neonatal bronchopulmonary dysplasia [10,11]. Their colonization induces cytokine production, initiating sequences leading to PTB and SA [4,5]. A recent study using polymerase chain reaction (PCR) detected genital *Mycoplasma* species in only 3.0% of amniotic fluid samples obtained for chromosomal analysis in asymptomatic pregnancies during early gestational weeks [12]. Given the high colonization rate of genital *Mycoplasma* species in the vagina [5], these results indicate that the organisms remain in the lower genital tract during early pregnancy and ascend into the choriodecidual space in later weeks, causing adverse pregnancy outcomes [13-15]. Therefore, screening for genital *Mycoplasma* species in the lower genital tract, followed by treatment, might be beneficial in preventing adverse pregnancy outcomes. However, screening for the organisms is not currently recommended, as previous studies did not show the reduction of PTB or neonatal outcomes by treating genital *Mycoplasma* species [6,16-18].

Nevertheless, there is an issue with these studies. A study should recruit individuals who receive treatment as the exposure group and those who do not receive treatment as the control group. However, many studies use individuals who are positive for genital *Mycoplasma* and receive treatment as the exposure group and those negative for the organisms as the control group [2,19,20]. This model produces selection bias when inferring treatment effects because it lacks appropriate control groups [21]. Kawakita et al. recently addressed this issue using a proportional hazards model and revealed no association between treatment and PTB [22]. However, their analysis did not account for follow-up examinations after the initial treatment. In addition, previous research suggests that treating co-infection of *Ureaplasma* and *Mycoplasma* species may be beneficial [20]. Therefore, we aimed to estimate the causal effect of treatment for genital *Mycoplasma* species on PTB and SA, incorporating both first and second treatments. We also evaluated treatment effects separately among women with co-infection of *Ureaplasma* and *Mycoplasma*.

## Materials and methods

Study design and setting

This was a retrospective cohort study of high-risk individuals for PTB at Hofheimer Hall, Norfolk, VA, a single academic institution, using data in Norfolk, USA, from January 2014 to December 2020. We evaluated the treatment effects for the genital *Mycoplasma* species on pregnancy outcomes, including PTB and SA, in singleton mothers who tested positive for the organisms. Exclusion criteria include individuals who tested negative for the organisms or multiple pregnancies. This study was approved by the Eastern Virginia Medical School Institutional Review Board (IRB approval No. 21-02-XX-0032).

Participants

In 2014, we implemented a quality improvement project in which* Ureaplasma* or *Mycoplasma* cervical culture was routinely obtained from high-risk mothers. We conducted a retrospective cohort study using this population. These risks included a history of PTB and PPROM, a history of two or more unexplained first-trimester SAs, cerclage placement, preterm contractions, and others such as cervical insufficiency (defined as cervical length <25 mm), heavy vaginal discharge, and a history of cerclage.

We screened for genital *Mycoplasma* species in the cervical external os of eligible individuals using a cotton swab. After screening, we initiated the first treatment, followed by re-screening and a second treatment. Although screening could be repeated up to three times, treatment was performed a maximum of two times.

The screening process was as follows: at their first visit, we assessed each patient’s history of PTB or PPROM and history of SA. For individuals without these histories, screening was deferred until they received a cerclage or reported preterm contractions later in pregnancy. Genital *Mycoplasma* species were screened using a specimen collection cotton swab placed within the external cervical os. This test detects genital *Mycoplasma* species, including *Mycoplasma genitalium*, *Mycoplasma hominis*, and *Ureaplasma* spp., by nucleic acid amplification. The treatment regimen for *Ureaplasma* only: azithromycin 500 mg orally on the first day, followed by 250 mg orally once daily for the next six days. For *Mycoplasma* only: clindamycin 300 mg orally twice daily for a total of seven days. For both *Ureaplasma* and *Mycoplasma*, a combination of the above treatments was applied [23]. We re-screened for genital *Mycoplasma* species following treatment and initiated re-treatment if the results remained positive.

Variables

The primary outcome was the risk difference (RD) in a combination of PTB and SA, defined as delivery between 22 weeks and 37 weeks of gestation and before 22 weeks of gestation, respectively. Therefore, the primary outcome means delivery before 37 weeks of gestation. The secondary outcomes included RD in PTB, PPROM, and chorioamnionitis. We included both spontaneous and medically indicated PTB. Intrauterine fetal demise (IUFD), defined as fetal death at any gestational week, was included as an outcome depending on the gestational week at delivery. PPROM was defined as premature rupture of membranes before 37 weeks of gestation. Chorioamnionitis was defined as clinical chorioamnionitis [24].

The causal pathway was visualized with the DAGitty program, version 3.1, which is available at https://www.dagitty.net/. In this study, we used the following variables as potential confounders: race, maternal age, pregestational diabetes, gestational diabetes, history of conization, cerclage placement, sexually transmitted infections (binary; positive for any of the following: *Chlamydia trachomatis*, *Neisseria gonorrhoeae*, *Trichomonas*, and syphilis), presence of preterm contractions at the time of screening, and gestational trimester at the screening (categorical: first, second, third). Race was composed of White, Black, and Other inidividuals in this study, and Others included Asian or Hispanic individuals. Race was used to adjust for confounding bias because it is associated with PTB [25]. These variables were used in estimating propensity scores for receiving treatment.

Statistical analysis

We defined the exposure group as individuals who tested positive for genital *Mycoplasma* species and received treatment, and the control group as individuals who tested positive but did not receive treatment. We analyzed the treatment effects separately for the first and second treatments. Additionally, we conducted subgroup analyses for the treatment effects by organism type (co-infection of both *Ureaplasma* and *Mycoplasma* species or either of them).

Inverse probability of treatment weighting (IPTW) was applied to estimate the adjusted RD. We used multivariate logistic regression models to estimate propensity scores for receiving the first and the second treatment separately, using the variables listed above. We then applied generalized estimating equations with individuals weighted by the propensity scores to estimate the causal RD and 95% CI. When there was no incidence of outcomes in either the exposure or control group, RD was not calculable and not reported.

Because information regarding the timing of treatments was not available, we assumed an interval of two weeks between screening and treatment and between treatment and re-screening. Additionally, we assumed a six-week interval when evaluating the effects of the second treatment. We excluded individuals who delivered within these periods, as they might not have received treatment before delivery. We also performed a sensitivity analysis by including this population.

Statistical significance was not used in this study. A recent statement from the American Statistical Association (ASA) recommended the careful use and evaluation of statistical significance [26]. All analyses were performed using R (version 4.4.2, R Foundation for Statistical Computing, Vienna, Austria).

## Results

Participants

**Figure 1:**
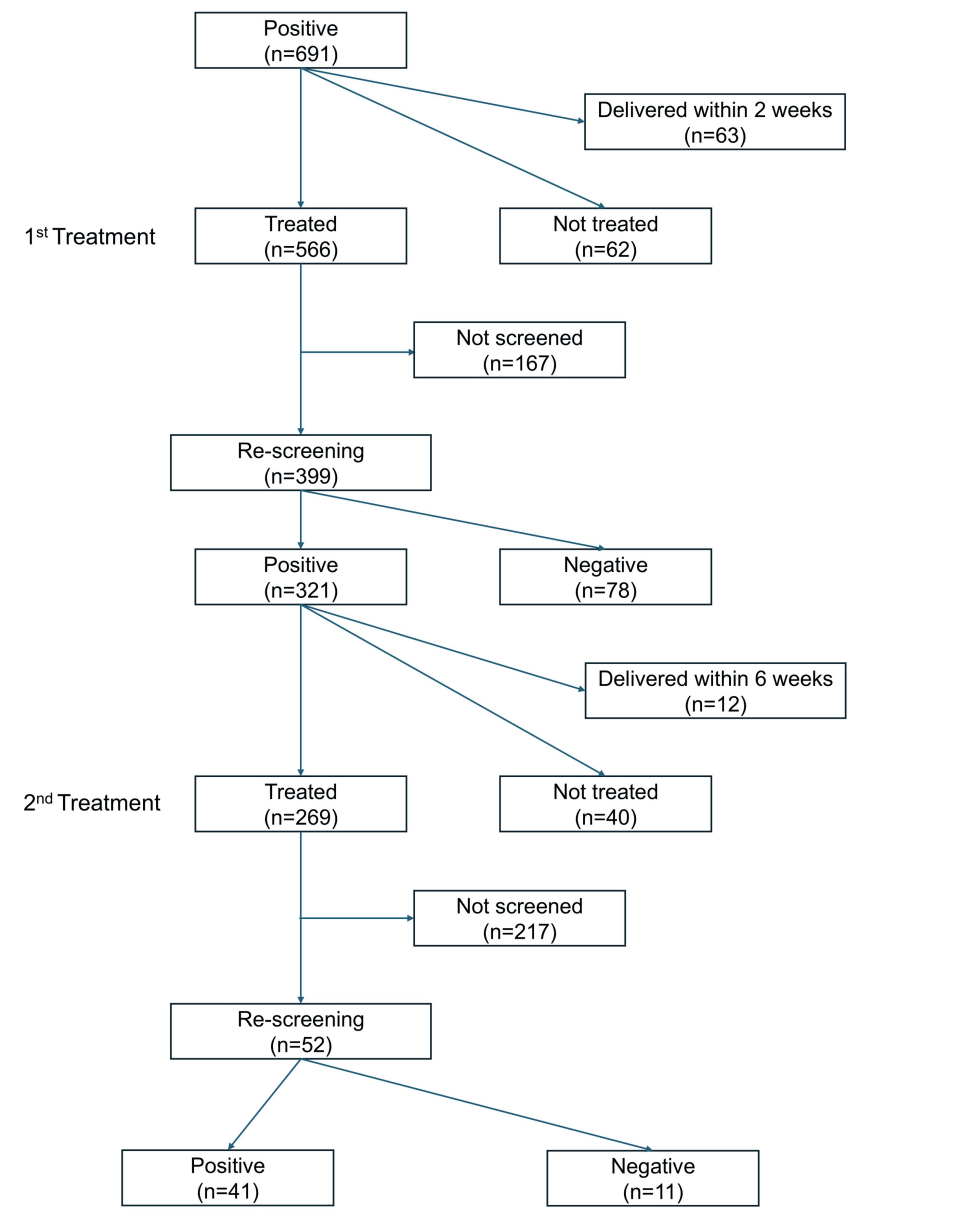
Study flow

Among the 691 individuals who tested positive for genital *Mycoplasma* species, 63 delivered within two weeks of the first screening and were therefore excluded. The remaining 628 were assigned for the first treatment, of whom 566 (90.1%) received treatment, and 62 (9.8%) did not. Individuals who received the first treatment (n = 566) proceeded to the second screening. Among these, 399 were re-screened for genital *Mycoplasma* species. Of these, 321 (80.5%) remained positive, while 78 (19.5%) tested negative. Among the 321 who remained positive, 12 delivered within six weeks of the first screening and were excluded from further treatment evaluation. Of the remaining individuals, 269 (87.1%) received the second treatment, while 40 (12.9%) did not. This participant flow was summarized in Figure 1. 

Descriptive data

We presented maternal demographics for the first treatment in Table 1 and for the second treatment in Table 2. The population was mostly represented by Black, multiparous, obese women. Black individuals were more likely to be assigned to the treatment group. While gestational diabetes was more frequent in the untreated group, pregestational diabetes and hypertensive disorders of pregnancy showed the opposite trend. Individuals with a history of conization using the loop electrosurgical excision procedure or the cold knife were more likely to be assigned to the untreated group. Most individuals in the study were screened for a history of PTB, PPROM, or two or more unexplained first-trimester SAs. Around 79.0% in the untreated group were screened for a history of PTB or PPROM, compared to 68.7% in the treated group. Both the treated and untreated groups were primarily screened during the first and second trimesters. In terms of infection type, 67.8% of the treated group were positive for *Ureaplasma* species, compared to 61.3% in the untreated group. Co-infection with *Ureaplasma* and *Mycoplasma* was observed in 27.6% of the treated group and 32.3% of the untreated group. Individuals infected with only *Mycoplasma* were small in both groups. This trend was also observed in the second treatment course (Table 2).

**Table 1:** Maternal demographics for the first treatment Maternal demographics for the first treatment are presented. ^1^n (%); median (min, max); ^2^GDM: gestational diabetes mellitus; ^3^HDP: hypertensive disorders of pregnancy; ^4^cervical procedure: history of conization using loop electrosurgical excision procedure or cold knife; ^5^STI: sexually transmitted infection, including *Chlamydia trachomatis*, *Neisseria gonorrhoeae*, *Trichomonas vaginalis*, and *Treponema pallidum* (syphilis); ^6^PTB: preterm birth; PPROM: preterm premature rupture of membranes; ^7^SA: spontaneous abortion.

Parameters	N	Overall N = 628^1^	Treated N = 566^1^	Untreated N = 62^1^
Race	628			
White		126 (20.1%)	111 (19.6%)	15 (24.2%)
Black		469 (74.7%)	425 (75.1%)	44 (71.0%)
Others		33 (5.3%)	30 (5.3%)	3 (4.8%)
Maternal Age (years)	628	29 (14, 50)	29 (14, 50)	30 (18, 41)
BMI	628	31 (17, 70)	31 (17, 70)	31 (18, 55)
Parity	628			
0		110 (17.5%)	99 (17.5%)	11 (17.7%)
1		215 (34.2%)	194 (34.3%)	21 (33.9%)
2		148 (23.6%)	136 (24.0%)	12 (19.4%)
≥3		155 (24.7%)	137 (24.2%)	18 (29.0%)
Previous Cesarean	628	183 (29.1%)	160 (28.3%)	23 (37.1%)
Smoking	628	58 (9.2%)	48 (8.5%)	10 (16.1%)
Illicit Drug Use	628	39 (6.2%)	34 (6.0%)	5 (8.1%)
Pregestational Diabetes	628	41 (6.5%)	40 (7.1%)	1 (1.6%)
GDM^2^	628	63 (10.0%)^2^	56 (9.9%)^2^	7 (11.3%)^2^
HDP^3^	628	81 (12.9%)^3^	75 (13.3%)^3^	6 (9.7%)^3^
Cervical Procedure^4^	628	44 (7.0%)^4^	38 (6.7%)^4^	6 (9.7%)^4^
Uterine Myoma	628	6 (1.0%)	6 (1.1%)	0 (0.0%)
History of Myomectomy	628	4 (0.6%)	3 (0.5%)	1 (1.6%)
STI^5^	628	60 (9.6%)^5^	54 (9.5%)^5^	6 (9.7%)^5^
Screening Criteria	628			
History of PTB or PPROM^6^		438 (69.7%)^6^	389 (68.7%)^6^	49 (79.0%)^6^
History of SA^7^		25 (4.0%)^7^	25 (4.4%)^7^	0 (0.0%)^7^
Cerclage		53 (8.4%)	46 (8.1%)	7 (11.3%)
Preterm Contractions		22 (3.5%)	20 (3.5%)	2 (3.2%)
Unknown		17 (2.7%)	16 (2.8%)	1 (1.6%)
Others		73 (11.6%)	70 (12.4%)	3 (4.8%)
Gestational Trimester at Screening	628			
First		201 (32.0%)	191 (33.7%)	10 (16.1%)
Second		374 (59.6%)	334 (59.0%)	40 (64.5%)
Third		53 (8.4%)	41 (7.2%)	12 (19.4%)
Genital *Mycoplasma*	628			
Both		176 (28.0%)	156 (27.6%)	20 (32.3%)
Mycoplasma		30 (4.8%)	26 (4.6%)	4 (6.5%)
Ureaplasma		422 (67.2%)	384 (67.8%)	38 (61.3%)

**Table 2:** Maternal demographics for the second treatment Maternal demographics for the first treatment are presented. ^1^n (%); median (min, max); ^2^GDM: gestational diabetes mellitus; ^3^HDP: hypertensive disorders of pregnancy; ^4^cervical procedure: history of conization using loop electrosurgical excision procedure or cold knife; ^5^STI: sexually transmitted infection, including *Chlamydia trachomatis*, *Neisseria gonorrhoeae*, *Trichomonas vaginalis*, and *Treponema pallidum* (syphilis); ^6^PTB: preterm birth; PPROM: preterm premature rupture of membranes; ^7^SA: spontaneous abortion.

Parameters	N	Overall N = 309^1^	Treated N = 269^1^	Untreated N = 40^1^
Race	309			
White		62 (20.1%)	53 (19.7%)	9 (22.5%)
Black		227 (73.5%)	196 (72.9%)	31 (77.5%)
Others		20 (6.5%)	20 (7.4%)	0 (0.0%)
Maternal Age (years)	309	29 (14, 45)	29 (14, 45)	31 (20, 42)
BMI	309	31 (17, 70)	31 (17, 70)	29 (19, 52)
Parity	309			
0		45 (14.6%)	41 (15.2%)	4 (10.0%)
1		108 (35.0%)	97 (36.1%)	11 (27.5%)
2		77 (24.9%)	67 (24.9%)	10 (25.0%)
≥3		79 (25.6%)	64 (23.8%)	15 (37.5%)
Previous Cesarean	309	86 (27.8%)	71 (26.4%)	15 (37.5%)
Smoking	309	28 (9.1%)	22 (8.2%)	6 (15.0%)
Illicit Drug Use	309	22 (7.1%)	19 (7.1%)	3 (7.5%)
Pregestational Diabetes	309	20 (6.5%)	17 (6.3%)	3 (7.5%)
GDM^2^	309	27 (8.7%)^2^	25 (9.3%)^2^	2 (5.0%)^2^
HDP^3^	309	37 (12.0%)^3^	34 (12.6%)^3^	3 (7.5%)^3^
Cervical Procedure^4^	309	19 (6.1%)^4^	17 (6.3%)^4^	2 (5.0%)^4^
Uterine Myoma	309	2 (0.6%)	2 (0.7%)	0 (0.0%)
History of Myomectomy	309	1 (0.3%)	1 (0.4%)	0 (0.0%)
STI^5^	309	29 (9.4%)^5^	25 (9.3%)^5^	4 (10.0%)^5^
Screening Criteria	309			
History of PTB or PPROM^6^		223 (72.2%)^6^	198 (73.6%)^6^	25 (62.5%)^6^
History of SA^7^		16 (5.2%)^7^	16 (5.9%)^7^	0 (0.0%)^7^
Cerclage		13 (4.2%)	11 (4.1%)	2 (5.0%)
Preterm Contractions		12 (3.9%)	9 (3.3%)	3 (7.5%)
Unknown		10 (3.2%)	7 (2.6%)	3 (7.5%)
Others		35 (11.3%)	28 (10.4%)	7 (17.5%)
Gestational Trimester at Screening	309			
First		122 (39.5%)	112 (41.6%)	10 (25.0%)
Second		176 (57.0%)	150 (55.8%)	26 (65.0%)
Third		11 (3.6%)	7 (2.6%)	4 (10.0%)
Genital *Mycoplasma*	309			
Both		88 (28.5%)	79 (29.4%)	9 (22.5%)
Mycoplasma		15 (4.9%)	12 (4.5%)	3 (7.5%)
Ureaplasma		206 (66.7%)	178 (66.2%)	28 (70.0%)

Main results

**Figure 2:**
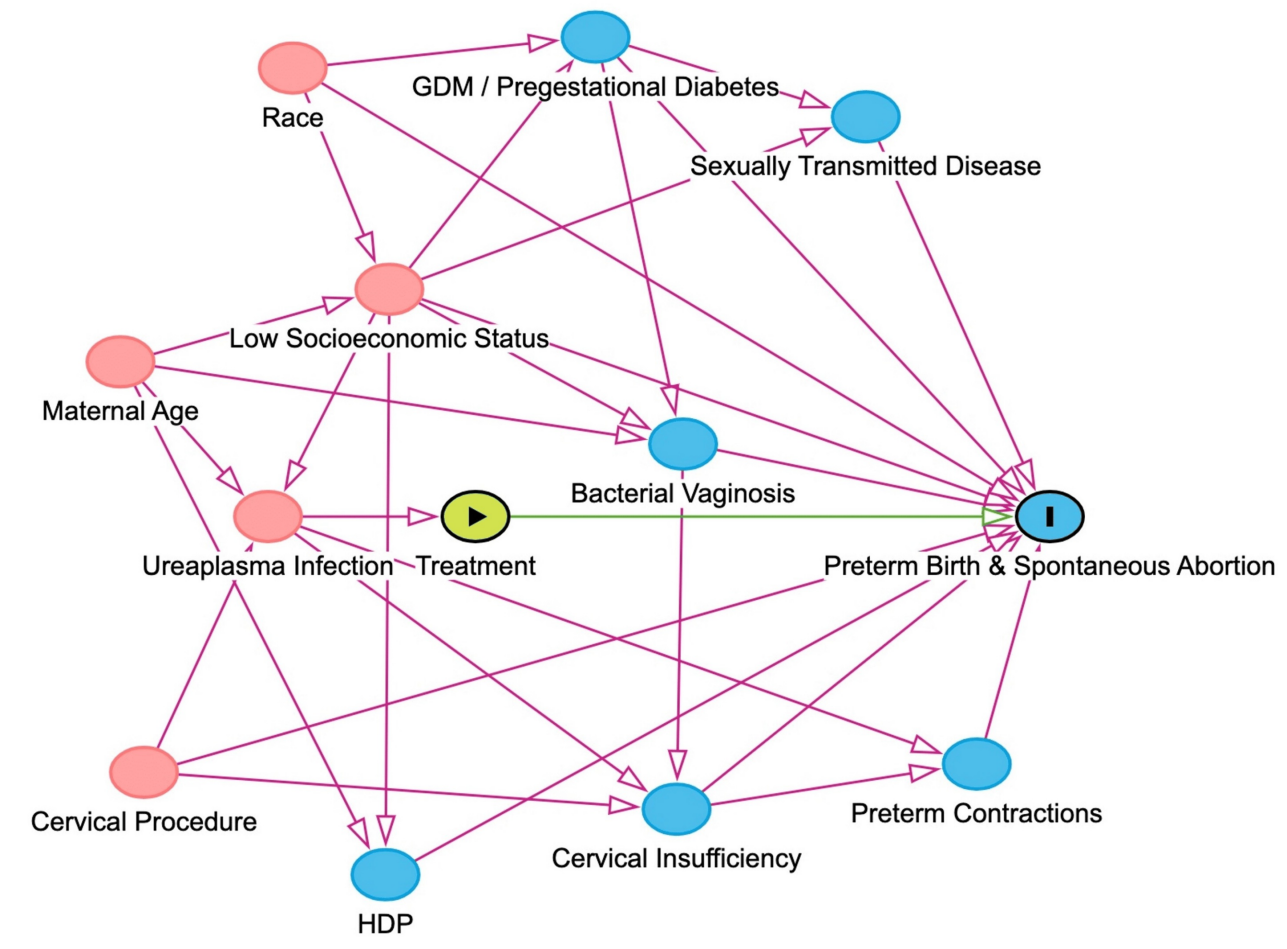
Causal pathway The causal pathway for preterm birth and spontaneous abortion is presented. HDP: hypertensive disorders of pregnancy; GDM: gestational diabetes mellitus

Figure 2 illustrates the causal pathway that this study employed. In this figure, bacterial vaginosis (BV) and socioeconomic status were not included in this database and were not adjusted in this study.

Table 3 displays the effects of treatment on primary and secondary outcomes. For the first treatment, PTB and SA occurred in 217/566 (38.3%) of the treated group and 24/62 (38.7%) of the untreated group. The adjusted RD and 95% confidence intervals, estimated using IPTW-weighted generalized estimating equations, were 0.037 (-0.104, 0.178). Among these, SA occurred in 11/566 (1.9%) of the treated group and 2/60 (3.3%) of the untreated group. For the second treatment, PTB and SA were observed in 89/269 (33.1%) of the treated group and 16/40 (40%) of the untreated group. The adjusted RD (95% CI) was -0.049 (-0.220, 0.123). SA occurred in 2/269 (0.74%) of the treated group and 1/40 (2.5%) of the untreated group. Secondary outcomes, including PTB, PPROM, and chorioamnionitis, were also presented in Table 3. PPROM occurred more frequently in the treated group for both the first and second treatments. Analysis was not performed for chorioamnionitis due to the small number of cases in the second treatment group. 

**Table 3:** Treatment effects This table shows adjusted outcomes, including individuals who delivered within the predefined weeks. ^a^RD: risk difference with the untreated group as reference; ^b^CI: confidence interval; ^c^PTB: preterm birth; ^d^SA: spontaneous abortion; ^e^PPROM: preterm premature rupture of membranes; ^f^: Not calculable due to lack of subjects in either group.

Treatment	Outcome	Treated (n, %)	Untreated (n, %)	Adjusted RD^a^ (95% CI^b^)
First treatment	PTB^c^ and SA^d^	217/566 (38.3%)	24/62 (38.7%)	0.037 (-0.104, 0.178)
	PTB	206/555 (37.1%)	22/60 (36.7%)	0.040 (-0.102, 0.182)
	PPROM^e^	69/566 (12.2%)	4/62 (6.5%)	0.052 (-0.034, 0.137)
	Chorioamnionitis	42/566 (7.4%)	4/62 (6.5%)	0.016 (-0.047, 0.079)
Second treatment	PTB and SA	89/269 (33.1%)	16/40 (40%)	-0.049 (-0.220, 0.123)
	PTB	87/267 (32.6%)	15/39 (38.5%)	-0.034 (-0.206, 0.137)
	PPROM	26/269 (9.7%)	3/40 (7.5%)	0.001 (-0.112, 0.114)
	Chorioamnionitis	15/269 (5.6%)	0/40 (0%)	-^f^

When individuals who delivered within the predefined intervals after the first screening (two weeks for the first treatment and six weeks for the second treatment) were included, RD estimates indicated positive treatment effects (Table 4). 

**Table 4:** Sensitivity analysis This table shows adjusted outcomes, including individuals who delivered within the predefined weeks. ^a^RD: risk difference with the untreated group as reference; ^b^CI: confidence interval; ^c^PTB: preterm birth; ^d^SA: spontaneous abortion; ^e^PPROM: preterm premature rupture of membranes; ^f^: Not calculable due to lack of subjects in either group.

Treatment	Outcome	RD^a^ (95% CI^b^)
First treatment	PTB^c^ and SA^d^	-0.129 (-0.259, 0.002)
	PTB	-0.088 (-0.226, 0.051)
	PPROM^e^	-0.087 (-0.184, 0.010)
	Chorioamnionitis	-0.045 (-0.116, 0.026)
Second treatment	PTB and SA	-0.129 (-0.292, 0.034)
	PTB	-0.118 (-0.283, 0.047)
	PPROM	-0.024 (-0.148, 0.101)
	Chorioamnionitis	-^f^

As for treatment of individuals infected with *Ureaplasma* only, RD (95% CI) was 0.049 (-0.144, 0.243) for the first treatment and -0.064 (-0.280, 0.153) for the second treatment. Treatment for co-infection yielded an RD (95% CI) of -0.005 (-0.274, 0.264) for the first treatment and -0.545 (-0.739, -0.350) for the second treatment. Treatment effects for *Mycoplasma* alone were not shown due to small sample sizes (Table 5). 

**Table 5:** Results by species This table shows the first and second treatment effects for genital *Mycoplasma* species overall, *Ureaplasma* only, and co-infection of both *Ureaplasma *spp. and *Mycoplasma* spp. ^a^RD: risk difference; ^b^CI: confidence interval.

Treatment	Genital *Mycoplasma* species RD^a^ (95% CI^b^)	*Ureaplasma* species RD (95% CI)	Co-infection RD (95% CI)
First treatment	0.037 (-0.104, 0.178)	0.049 (-0.144, 0.243)	-0.005 (-0.274, 0.264)
Second treatment	-0.049 (-0.220, 0.123)	-0.064 (-0.280, 0.153)	-0.545 (-0.739, -0.350)

## Discussion

Principal findings

Regarding PTB and SA, the result indicated that the treatment appeared ineffective at the first screening but beneficial at the second. The results for PPROM and chorioamnionitis were difficult to interpret due to the limited number of events in this cohort. However, when focusing on the treatment effects for co-infection with *Ureaplasma* and *Mycoplasma*, we observed small but positive treatment effects for PTB and SA. The treatment response was low, with approximately 80% of individuals remaining positive for genital *Mycoplasma* species after treatment.

Results in the context of the literature

Genital *Mycoplasma* species are highly prevalent in the lower genital tract of reproductive-age women [19,27]. Although often regarded as low-virulence commensals, their association with adverse pregnancy outcomes, particularly PTB and SA, has gained increasing attention, especially when they are detected in the amniotic fluid [10,28].

Recent meta-analyses have suggested an increased risk of PTB and SA in colonized individuals, particularly in the presence of abnormal vaginal flora [7,10]. However, these studies are biased by heterogenicity in sampling methods: some studies collect specimens from the vagina, while others use amniotic fluid. In addition, potential confounders such as BV are often not fully adjusted.

Additionally, some cohort studies have challenged this association. Rauh et al. examined the presence of genital *Mycoplasma* species in individuals with cervical insufficiency and found no difference in gestational age at delivery regardless of infection status [19]. Similarly, a prospective cohort study by Cunha et al. also concluded that infection with genital *Mycoplasma* species was not a risk factor for PTB [2]. Therefore, the association remains controversial.

Regarding treatment strategies, azithromycin is effective against genital *Mycoplasma *species in vitro [29]. However, in clinical settings, the treatment of genital *Mycoplasma* species in the vagina has not been sufficiently explored. One randomized controlled trial (RCT) investigated the use of erythromycin for treating genital *Mycoplasma *species in the vagina and found no clear differences in the incidence of low-birth-weight infants [16].

Our study addressed the treatment of genital *Mycoplasma *species in the vagina and suggested potential benefits in treating co-infection with *Ureaplasma* spp. and *Mycoplasma *spp. Vouga et al. also demonstrated the possible benefits of treating co-infection, as it may reduce PTB and neonatal morbidity [20]. Genital *Mycoplasma* species increase vaginal pH through ammonia production mediated by urea hydrolysis from *Ureaplasma* and arginine from* Mycoplasma, *which alkalizes the vaginal environment. This can facilitate infections with other bacteria, such as those associated with BV [6,30]. One possible explanation for the observed positive treatment effects in this study is that eradicating both species may help restore the vaginal flora, thereby reducing the risk of PTB and SA.

Clinical implications

Although current evidence supports the use of antibiotic therapy in cases of confirmed intra-amniotic infection and inflammation, its role in managing asymptomatic vaginal colonization remains unestablished due to limited supporting evidence. Thus, routine screening and treatment for asymptomatic genital *Mycoplasma* infections during pregnancy are not recommended by current guidelines, such as those issued by the European STI Guidelines Board [17]. This is due to the high carriage rate, variable pathogenicity, and concerns over antimicrobial resistance. However, as our findings suggest, in cases of co-infection with *Ureaplasma* and *Mycoplasma*, treatment could be considered, as it may help prevent PTB and SA.

Strengths and limitations

A key strength of our study was that we clearly defined individuals who were positive for genital *Mycoplasma* species and treated as the exposure group and untreated individuals as the control. We also evaluated the effects of a second treatment and assessed treatment response using PCR.

Nevertheless, this study has several limitations. First, because this was a retrospective cohort study based on the population in our quality improvement project, selection bias is a concern. The individuals did not receive the treatment for various reasons, including declining therapy or being unreachable when treatment was indicated. These factors inevitably introduced selection bias. Second, a substantial portion of individuals did not undergo follow-up testing after treatment, which limited our ability to confirm eradication of the organisms. Moreover, we were unable to differentiate between various *Ureaplasma* species due to data limitations. Recent evidence suggests that *Ureaplasma parvum* is more strongly associated with PTB than *Ureaplasma urealyticum* [5,31]. Therefore, treatment effects may have been more informative if the analysis had been restricted to specific subspecies. Moreover, previous studies have highlighted the importance of accounting for BV as a confounding factor [7]. Socioeconomic status was also an important factor in pregnancy outcomes [2]. However, because our dataset did not include these variables, we were unable to adjust them. Finally, as with any single-center cohort study, the generalizability of our findings is limited. Our cohort was primarily composed of Black, multiparous, obese women at high risk for PTB. Therefore, caution is warranted when applying these results to other populations, such as women of different racial backgrounds or nulliparous women.

## Conclusions

In this study, we examined the treatment effects of genital *Mycoplasma* among high-risk populations, and we found no clear evidence to support routine treatment for genital *Mycoplasma* species in the vagina. However, there may be potential treatment benefits for individuals co-infected with *Ureaplasma* spp. and *Mycoplasma* spp. Future research should investigate treatment effects across different subspecies and patterns of co-infection. Additionally, potential confounders, such as BV, should be carefully adjusted in future research.
